# Malaria Among Members of the U.S. Armed Forces, 2024

**Published:** 2025-04-20

## Abstract

Malaria infection remains a potential health threat to U.S. service members located in or near endemic areas due to duty assignments, participation in contingency operations, or personal travel. In 2024, a total of 30 active and reserve component service members were diagnosed with or reported to have malaria, a 23.1% decrease from the 39 cases identified in 2023. Over half of U.S. service member malaria cases in 2024 were caused by *Plasmodium falciparum* (56.7%, n=17), followed by unspecified types of malaria (33.3%, n=10) and *P. vivax* (10.0%, n=3). Malaria cases were diagnosed or reported from 18 different medical facilities in the U.S., Germany, Africa, Japan, Middle East, and South Korea. Of the 27 cases with a known location of diagnosis, 11 (40.7%) were reported or diagnosed outside the U.S.

Malaria, a life-threatening disease spread to humans through the bite of *Anopheles* mosquitoes, is transmitted mostly in tropical countries. ^1^ The World Health Organization (WHO) estimated 263 million malaria cases (incidence rate of 60.4 cases per 1,000 population at risk) in 2023 and 597,000 deaths (mortality rate of 13.7 per 100,000) within 83 endemic countries. Of those 83 countries with known malaria cases, 29 countries accounted for nearly 95% of cases and 96% of deaths. ^2^ The 5 countries with the greatest estimated burdens of malaria are Nigeria (26%), Democratic Republic of the Congo (13%), Uganda (5%), Ethiopia (4%), and Mozambique (4%). ^2^

Four species of *Plasmodium* account for the most significant burdens of malaria disease in humans: *P. falciparum, P. vivax, P. malariae* , and *P. ovale. P. falciparum* is the most dangerous form of malaria, accounting for over 90% of malaria-related deaths. ^3^ While *P. falciparum* is most prevalent in Africa, *P. vivax* is the most widely distributed parasite species geographically, with relatively high prevalences of infection in the regions of Southeast Asia, the western Pacific, and eastern Mediterranean, as well as less densely populated areas of the Americas. ^4^

Malaria is not endemic in the U.S. but remains a significant threat to its military service members deployed to tropical and subtropical regions. This risk to U.S. service members is due to operational constraints, lack of compliance with available preventive measures, in addition to continuing emergence of drug-resistant malarial parasites. ^5^ The U.S. Armed Forces have long maintained policies and prescribed measures effective against vector-borne diseases such as malaria, including chemoprophylactic drugs, permethrin-impregnated uniforms and bed nets, and topical insect repellents. During planning for overseas military operations, geographically-associated presence or absence of malaria risk is usually known and can be anticipated, but implementation of preventive measures can be complex and dependent upon individual adherence to personal protective measures. When cases and outbreaks of malaria occur, they are generally due to poor adherence to chemoprophylaxis and other personal preventive measures. ^6 - 9^

## What are the new findings?

This report documents a total of 30 malaria cases in 2024, a 23.1% decrease from 39 cases in 2023, mainly due to declines in Africa and other or unspecified locations. As in 2023, *Plasmodium falciparum* continues to constitute over half of new malaria cases (n=17, 56.7%) among active and reserve component U.S. service members.

## What is the impact on readiness and force health protection?

Malaria poses a risk for service members deployed to endemic regions or during travel to such areas for personal reasons. *P. falciparum* , the most dangerous malaria strain, with a high risk of serious sequelae, including death, was diagnosed in more than half of cases in 2024. This finding emphasizes the need for continued preventive mea sures and heightened awareness of potential diagnostic challenges, particularly in areas where *P. falciparum* is endemic.

Since 1999, *MSMR* has published regular updates on malaria incidence among U.S. service members. *MSMR* 's sustained focus on malaria reflects both historical trends about this mosquito-borne disease and the continuing threat it poses to military readiness and service member health. This update describes the epidemiological patterns of malaria incidence among service members in the active and reserve components of the U.S. Armed Forces from 2015 through 2024.

## Methods

The surveillance population for this report includes service members of the U.S. Army, Navy, Air Force, Marine Corps, Space Force, and Coast Guard. The surveillance period was January 1, 2015 through December 31, 2024. Records from the Defense Medical Surveillance System (DMSS) were searched to identify qualifying evidence of a malaria diagnosis from reportable medical events (RMEs), hospitalizations, outpatient encounters (in military and non-military facilities), and laboratory results from military facilities.

Case definition criteria for malaria included either 1) an RME record of confirmed malaria, 2) a hospitalization record with a primary diagnosis of malaria, 3) a hospitalization record with a non-primary diagnosis of malaria due to a specific *Plasmodium* species, 4) a hospitalization record with a non-primary diagnosis of malaria plus a diagnosis of anemia, thrombocytopenia, and related conditions, or malaria-complicating pregnancy in any diagnostic position, 5) a hospitalization record with a non-primary diagnosis of malaria plus diagnoses of signs or symptoms consistent with malaria in each diagnostic position preceding malaria, or 6) a positive malaria antigen test plus an outpatient record with a diagnosis of malaria in any diagnostic position within 30 days of the specimen collection date. ^10^ The relevant International Classification of Diseases, 9th and 10th Revision (ICD-9 / ICD-10) codes used to identify cases are shown in **Table 1** .

**TABLE 1.** ICD-9 and ICD-10 Diagnosis Codes Used to Define Malaria Cases from Inpatient Encounters (Hospitalizations)

	ICD-9	ICD-10
Malaria *Plasmodium* species		
*P. falciparum*	84.0	B50
*P. vivax*	84.1	B51
*P. malariae*	84.2	B52
*P. ovale*	84.3	B53.0
Unspecified	84.4, 84.5, 84.6, 84.8, 84.9	B53.1, B53.8, B54
Anemia	280–285	D50–D53, D55–D64
Thrombocytopenia	287	D69
Malaria complicating pregnancy	647.4	O98.6
Signs, symptoms, or other abnormalities consistent with malaria	276.2, 518.82, 584.9, 723.1, 724.2, 780.0, 780.01, 780.02, 780.03, 780.09, 780.1, 780.3, 780.31, 780.32, 780.33, 780.39, 780.6, 780.60, 780.61, 780.64, 780.65, 780.7, 780.71, 780.72, 780.79, 780.97, 782.4, 784.0, 786.05, 786.09, 786.2, 786.52, 786.59, 787.0, 787.01, 787.02, 787.03, 787.04, 789.2, 790.4	E87.2, J80, M54.2, M54.5, N17.9, R05, R06.0, R06.89, R07.1, R07.81, R07.82, R07.89, R11*, R16.1, R17, R40*, R41.0, R41.82, R44*, R50*, R51, G44.1, R53*, R56*, R68.0, R68.83, R74.0

Abbreviations: ICD-9, International Classification of Diseases, 9th Revision; ICD-10, International Classification of Diseases, 10th Revision; *P., plasmodium* .

This analysis restricted each service member to 1 episode of malaria per 365-day period. When multiple records documented a single episode, the date of the earliest record was considered the date of clinical onset. Records within 30 days of the clinical onset date were reviewed for evidence of a *Plasmodium* species.

Presumed locations of malaria acquisition were estimated with a hierarchical algorithm: 1) cases diagnosed in a malariaendemic country were considered acquired in that country, 2) RMEs that listed exposures to malaria-endemic locations were considered acquired in those locations, 3) RMEs not listing exposures to malaria-endemic locations but were reported from installations in malaria-endemic locations were considered acquired in those locations, 4) cases diagnosed among service members during or within 30 days of deployment or assignment to a malaria-endemic country were considered acquired in that country, and 5) cases diagnosed among service members deployed or assigned to a malaria-endemic country within 2 years before diagnosis were considered acquired in those countries. All remaining cases were considered to have acquired malaria in unknown locations.

## Results

**FIGURE 1.**
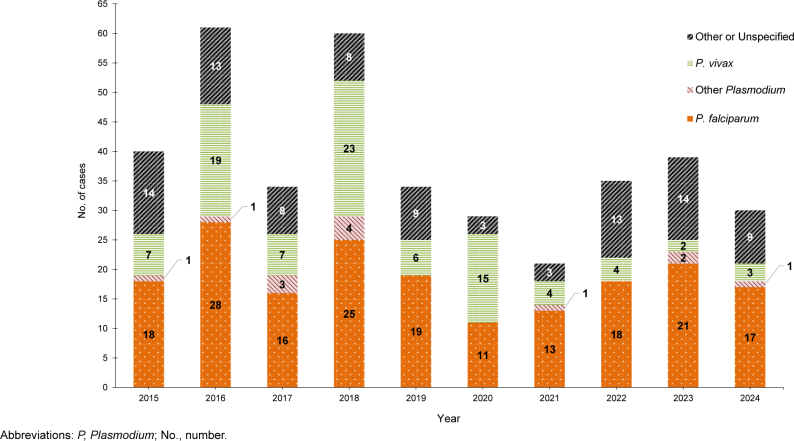
Numbers of Malaria Cases by Species and Calendar Year of Diagnosis or Report, Active and Reserve Components, U.S. Armed Forces, 2015–2024

In 2024, a total of 30 U.S. service members were diagnosed with, or reported to have, malaria **(Table 2)** , resulting in a rate of 1.5 per 100,000 persons (data not shown). The annual total for 2024 represents a 23.1% decrease in malaria cases from the 39 cases reported in 2023 **(Figure 1)** .

**TABLE 2.** Malaria Cases by *Plasmodium* Species and Selected Demographic Characteristics, U.S. Armed Forces, 2024

	*P. vivax*	*P. falciparum*	Other or Unspecified	Total		DMSS AC Reference Population ^a^	
	No.	No.	No.	No.	%	No.	%
Total	3	17	10	30	100.0	2,055,342	100.0
Sex							
Male	2	17	10	29	96.7	1,656,109	80.6
Female	1	0	0	1	3.3	399,233	19.4
Age group, y							
<20	0	0	0	0	0.0	124,545	6.1
20–24	1	3	3	7	23.3	556,300	27.1
25–29	0	1	1	2	6.7	447,423	21.8
30–34	1	7	4	12	40.0	341,310	16.6
35–39	1	5	2	8	26.7	281,151	13.7
40–44	0	0	0	0	0.0	171,209	8.3
45–49	0	1	0	1	3.3	74,255	3.6
50 +	0	0	0	0	0.0	59,149	2.9
Race and ethnicity							
White, non-Hispanic	1	3	7	11	36.7	1,101,662	53.6
Black, non-Hispanic	1	13	3	17	56.7	331,528	16.1
Hispanic	0	0	0	0	0.0	387,682	18.9
Other	1	1	0	2	6.7	234,470	11.4
Component							
Active	3	14	9	26	86.7	1,294,111	63.0
Reserve / Guard	0	3	1	4	13.3	761,231	37.0
Service							
Army	1	12	4	17	56.7	938,452	45.7
Navy	0	2	3	5	16.7	379,580	18.5
Air Force	0	2	0	2	6.7	481,381	23.4
Marine Corps	2	1	3	6	20.0	200,621	9.8
Coast Guard	0	0	0	0	0	45,940	2.2
Space Force	0	0	0	0	0	9,368	0.5

Abbreviations: *P., Plasmodium* ; DMSS, Defense Medical Surveillance System; AC, all components; y, years.

a Data Source: Defense Medical Surveillance System (DMSS) as of Feb. 19, 2025 prepared by the Defense Health Agency.

Fifteen (50.0%) of the 30 cases in 2024 were identified from RME records. The remaining 15 cases were identified through additional case definition criteria: 11 cases from hospitalization records and 4 cases from a positive malaria antigen test plus an outpatient record with a diagnosis of malaria in any diagnostic position within 30 days of specimen collection date.

As in previous years, the majority of U.S. military members diagnosed with malaria in 2024 were men (96.7%), members of the active component (86.7%), and in the Army (56.7%). No cases were reported in the Space Force or Coast Guard. Non-Hispanic Black service members and those aged 30-34 years accounted for the most cases of malaria (56.7% and 40.0%, respectively) **(Table 2)** .

Examination of the 15 malaria case records reported as RMEs revealed that 6 of the case exposures were classified as deployment-related, 6 as non-duty-related, 2 as duty-related but not deployment-related, and 1 case was missing exposure classification. All of the 6 non-duty exposure cases were considered to have been acquired in Africa (data not shown).

**FIGURE 2.**
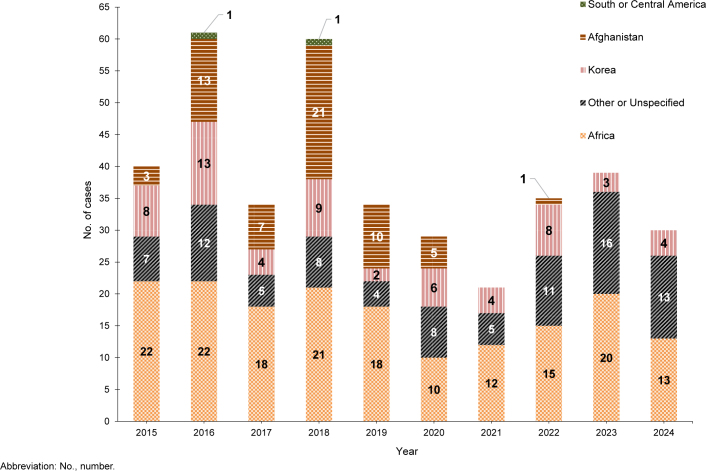
Numbers of Malaria Cases by Location of Acquisition, Active and Reserve Components, U.S. Armed Forces, 2015–2024

During the 2015-2024 surveillance period, malaria cases acquired in Africa (n=171, 44.6%) and other or unspecified locations (n=89, 23.2%) accounted for the largest numbers, followed by Korea (n=61, 15.9%), Afghanistan (n=60, 11.7%), and South and Central America (n=2, 0.5%) **(Figure 2)** . The annual percentages of cases associated with Africa had the greatest variability, ranging from 34.5% in 2020 to 60.0% in 2021. Malaria cases were diagnosed or reported in 2024 from 18 different medical facilities in the U.S. (n=12), Germany (n=2), Africa (n=1), Japan (n=1), Middle East (n=1), and South Korea (n=1) **(Table 3)** .

**TABLE 3.** Number of Malaria Cases by Geographic Location of Diagnosis or Report and Presumed Location of Acquisition, Active and Reserve Components, U.S. Armed Forces, 2024

Location Where Diagnosed or Reported	Korea	Africa	South or Central America	Other or Unknown Location	Total	
	No.	No.	No.	No.	No.	%
William Beaumont AMC, Fort Bliss, TX	0	1	0	3	4	13.3
NH Okinawa, Japan	1	0	0	3	4	13.3
Darnall AMC, Fort Cavazos, TX	0	2	0	0	2	6.7
Expeditionary Medical Facility, Djibouti	0	2	0	0	2	6.7
Brian D. Allgood ACH, Pyeongtaek, South Korea	1	1	0	0	2	6.7
NH Camp Pendleton, CA	0	1	0	0	1	3.3
NMC San Diego, CA	0	1	0	0	1	3.3
Evans Carson ACH, Fort Carson, CO	0	0	0	1	1	3.3
96th Medical Group, Eglin AFB, FL	0	0	0	1	1	3.3
Winn ACH, Fort Stewart, GA	0	1	0	0	1	3.3
Walter Reed National Military Medical Center, MD	0	0	0	1	1	3.3
Fort Meade Medical Department, MD	0	0	0	1	1	3.3
Reynolds AHC, Fort Sill, OK	1	0	0	0	1	3.3
Madigan AMC, Fort Lewis, WA	1	0	0	0	1	3.3
Naval Station Norfolk Branch Health Clinic, VA	0	0	0	1	1	3.3
Landstuhl Regional Medical Center, Germany	0	1	0	0	1	3.3
86th Medical Group, Ramstein Air Base, Germany	0	1	0	0	1	3.3
NBHC Naval Support Activity, Bahrain	0	1	0	0	1	3.3
Location not reported	0	1	0	2	3	10.0
Total	4	13	0	13	30	100

Abbreviations: No., number; AMC, Army Medical Center; ACH, Army Community Hospital; AHC, Army Health Clinic; AMC, Army Medical Center; AFB, Air Force Base; NH, Naval Hospital; NMC, Naval Medical Center.

**FIGURE 3.**
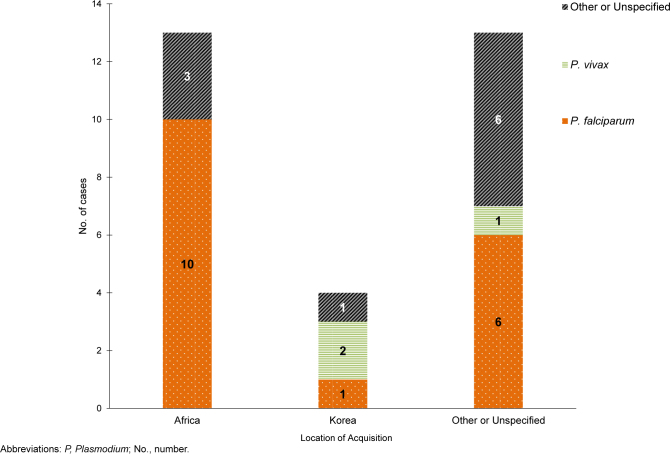
Numbers of Malaria Cases by Species Type and Location of Acquisition, Active and Reserve Components, U.S. Armed Forces, 2024

Over half of U.S. service member malaria cases in 2024 were caused by *P. falciparum* (56.7%, n=17). Of the 13 cases not attributed to *P. falciparum* , 3 (10.0%) were caused by *P. vivax* , while 10 were associated with other or unspecified types of malaria (33.3%) **(Figure 3)** .

In 2024, most cases acquired in Africa (n=13) were caused by *P. falciparum* (76.9%, n=10) **(Figure 3)** . The 13 malaria cases acquired in Africa were linked to several countries, including Djibouti (n=3), Cameroon (n=2), Nigeria (n=2), Chad (n=1), Gabon (n=1), Ghana (n=1), Senegal (n=1), and Uganda (n=1); 1 case was associated with an unknown African location (data not shown).

Over the past 10 years, malaria caused by *P. falciparum* has accounted for the largest number of cases (n=186, 48.6%) followed by other or unspecified species (n=94, 24.5%), *P. vivax* (n=90, 23.5%), and other *Plasmodium* species (n=13, 3.8%). The annual percentages of cases attributed to *P. vivax* from 2015 through 2024 showed the greatest variability, ranging from 5.1% in 2023 to 51.7% in 2020 (data not shown).

**FIGURE 4.**
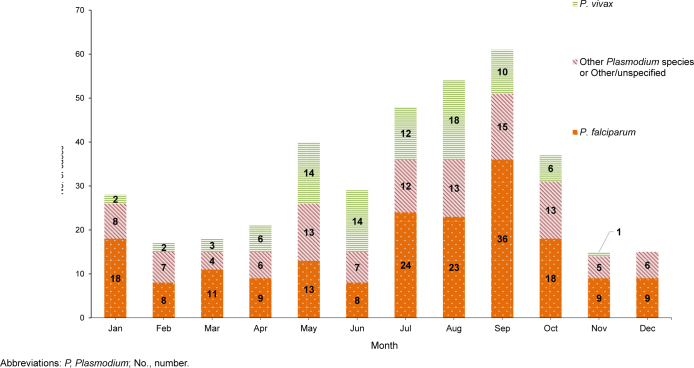
Cumulative Numbers of Malaria Cases by Species Type and Month of Clinical Presentation or Diagnosis, Active and Reserve Components, U.S. Armed Forces, 2015–2024

Between 2015 and 2024, most non- *P. vivax* malaria cases (66.1%) were diagnosed or reported during the 6 months from the Northern Hemisphere middle of spring through the middle of autumn (i.e., May-October) **(Figure 4)** . During the 10-year surveillance period, the proportions of non- *P. vivax* malaria cases diagnosed or reported from May through October varied by region of acquisition: Afghanistan (86.4%, n=19 / 22), Korea (79.2%, n=19 / 24), Africa (68.5%, n=113 / 165), and South and Central America (50.0%, n=1 / 2) (data not shown).

## Discussion

The 30 active and reserve component service members diagnosed with or reported to have malaria in 2024 represent a 23.1% decrease from the 39 cases reported in 2023. This decline may be attributed to effective countermeasures such as chemoprophylaxis and insecticide-treated uniforms or decreased risk of U.S. military personnel in areas of high malaria transmission. The most substantial decline in malaria cases reported from 2020 through 2021 may be attributed to progressive withdrawal of U.S. personnel from Afghanistan, along with restrictions on international travel due to the COVID-19 pandemic.

In 2024, *P. falciparum* persisted in more than half of U.S. service member malaria cases, demonstrating the need for continued focus on disease prevention, given its severity and mortality. The persistent burden of *falciparum* malaria acquired in Africa also emphasizes the importance of timely diagnostics for service members in deployed settings. The possibility of false negative results for *P. falciparum* on rapid diagnostic tests (RDTs) favored by units in resource-limited or austere locations was noted in 2019. ^11^ Since then, the emerging prevalence of mutant pfhrp2/3-deleted *P. falciparum* parasites has been described in parts of U.S. Southern Command (SOUTHCOM) and Africa Command (AFRICOM), highlighting the risk of hrp2-based rapid diagnostic tests as an unsuitable diagnostic tool for malaria in many countries. ^12^ In 2019, WHO outlined new recommendations to use non-HRP2-based RDTs when the prevalence of pfhrp2/3 deletions that cause false-negative results exceeds 5% in the specified geographic area for malaria risk. ^13^ These recommendations present a need for continued surveillance on the frequency and distribution of these mutant parasites where service members may deploy, as well as the development of alternative RDTs. ^14^

Malaria continues to present a medical concern for service members traveling to endemic regions while on leave, as 40% of malaria cases in RMEs in 2024 occurred during non-duty travel. For service members traveling to malaria-endemic regions, pre-travel chemoprophylaxis should be emphasized; however, prescribing practices vary among Military Health System (MHS) and civilian health care providers. ^14^ While force health protection policy plays a major role in standardizing chemopro-phylaxis regimens that may be indicated for a mission plan, ^15^ solutions are needed to extend risk management and prevention policies beyond large-scale deployment conditions. ^14^

This report does not assess prescribed chemoprophylaxis adherence, but several studies document low adherence and inadequate chemoprophylaxis during periods of deployment or travel to endemic regioms. ^16 , 17^ In 2018, the CDC assessed 38 U.S. military personnel malaria cases using the National Malaria Surveillance System (NMSS) and National Notifiable Diseases Surveillance System (NNDSS), finding that 25 (65.8%) personnel members received any form of prophylaxis; of those, 7 (28.0%) took all doses of a correct regimen. ^18^ Only half of the malaria cases among active and reserve component U.S. service members in 2024 identified in this report were from RME records, hindering full assessment of chemoprophylaxis use and adherence.

Seasonality patterns should be considered in force health protection plans for optimal vector control and drug-based intervention strategies. ^19^ Non- *P. vivax* malaria case seasonality in this report is compatible with a presumption of greatest risk of malaria acquisition from May through October in temperate, climatic zones of the Northern Hemisphere. Rain-fall and temperature are also significant factors for malaria seasonality; rainfall postpones onset of malaria transmission only in areas with high seasonal precipitation from September through November, as in sub-Saharan Africa; otherwise, malaria may be transmitted all year. ^20^

Limitations to this report should be considered when interpreting its findings. Malaria case reporting, especially for reserve components and non-deployment exposures, is likely incomplete, contributing to under-estimation of rates; some cases treated in deployed or non-U.S. military medical facilities may not have been reported or otherwise ascertained at time of analysis. Malaria diagnoses documented only in outpatient settings without confirmatory testing and not reported as RMEs were not included in this report. Geographic location of malaria acquisition was estimated from reported information, with some cases reporting exposures in multiple malaria-endemic areas and others with no relevant exposure information. Personal travel or deployment to malaria-endemic countries was not documented unless specified in RMEs. Limited information on species type in RME records emphasizes the need for more complete attention to documentation of reportable conditions.

*MSMR* annually publishes malaria cases identified through comprehensive surveillance—evaluation of RMEs, hospitalization records, and laboratory results generated from the MHS—to inform force health protection policy. Malaria infection remains a potential health threat to U.S. service members within or near endemic areas due to duty assignment, contingency operations, or personal travel.
